# The Diverse Cellular and Animal Models to Decipher the Physiopathological Traits of *Mycobacterium abscessus* Infection

**DOI:** 10.3389/fcimb.2017.00100

**Published:** 2017-04-04

**Authors:** Audrey Bernut, Jean-Louis Herrmann, Diane Ordway, Laurent Kremer

**Affiliations:** ^1^IRIM (ex-CPBS)-UMR 9004, Centre National de la Recherche Scientifique (CNRS), Infectious Disease Research Institute of Montpellier, Université de MontpellierMontpellier, France; ^2^UMR 1173, Institut National de la Santé et de la Recherche Médicale, Université de Versailles Saint-Quentin-en-YvelinesMontigny-le-Bretonneux, France; ^3^Mycobacteria Research Laboratory, Department of Microbiology, Immunology and Pathology, Colorado State UniversityFort Collins, CO, USA; ^4^Institut National de la Santé et de la Recherche Médicale, IRIMMontpellier, France

**Keywords:** *Mycobacterium abscessus*, infection, zebrafish, mouse, macrophage, amoeba, chemotherapy, cystic fibrosis

## Abstract

*Mycobacterium abscessus* represents an important respiratory pathogen among the rapidly-growing non-tuberculous mycobacteria. Infections caused by *M. abscessus* are increasingly found in cystic fibrosis (CF) patients and are often refractory to antibiotic therapy. The underlying immunopathological mechanisms of pathogenesis remain largely unknown. A major reason for the poor advances in *M. abscessus* research has been a lack of adequate models to study the acute and chronic stages of the disease leading to delayed progress of evaluation of therapeutic efficacy of potentially active antibiotics. However, the recent development of cellular models led to new insights in the interplay between *M. abscessus* with host macrophages as well as with amoebae, proposed to represent the environmental host and reservoir for non-tuberculous mycobacteria. The zebrafish embryo has also appeared as a useful alternative to more traditional models as it recapitulates the vertebrate immune system and, due to its optical transparency, allows a spatio-temporal visualization of the infection process in a living animal. More sophisticated immunocompromised mice have also been exploited recently to dissect the immune and inflammatory responses to *M. abscessus*. Herein, we will discuss the limitations, advantages and potential offered by these various models to study the pathophysiology of *M. abscessus* infection and to assess the preclinical efficacy of compounds active against this emerging human pathogen.

## Introduction

*Mycobacterium abscessus* (*Mabs*) is a rapidly-growing mycobacterial species, regarded as an important pathogen responsible for a wide array of clinical manifestations in humans, ranging from cutaneous infections to severe chronic pulmonary infections, usually encountered in immunocompromised and in cystic fibrosis (CF) patients (Griffith et al., 1993; Olivier et al., 2003; Jönsson et al., 2007; Roux et al., 2009; Leão et al., 2010; Qvist et al., 2015; Bryant et al., 2016). *Mabs* is also responsible for nosocomial and iatrogenic infections and has been reported to induce lesions in the central nervous system in immunocompetent patients with a history of trauma, otolaryngological diseases, neurosurgery, or disseminated disease in a patient with end-stage renal disease under steroid therapy (Talati et al., 2008; Lee et al., 2012). Despite being a rapidly-growing mycobacteria (RGM), *Mabs* shares also several pathophysiological features with the slow-growing *Mycobacterium tuberculosis*, the causative agent of tuberculosis. This includes the ability to persist within granulomatous structures and to generate pulmonary caseous lesions (Tomashefski et al., 1996; Medjahed et al., 2010). Alarming is the natural resistance of *Mabs* to most antitubercular drugs, making these infections particularly long, difficult to treat and associated with a significant therapeutic failure rate (Ferro et al., 2016). *Mabs* manifests as either a smooth (S) or a rough (R) colony morphotype that can result in different clinical outcomes. Epidemiological studies indicate that the presence of R variants are associated with the most severe cases of pulmonary infections which can persist for years (Jönsson et al., 2007; Catherinot et al., 2009). The morphological aspect of S and R variants relies on the presence or absence of surface-associated glycopeptidolipids (GPL), respectively (Howard et al., 2006). However, our knowledge of the pathophysiological characteristics and mechanisms governing virulence of the R or S variants has long been obscured by the lack of animal models that are permissive to *Mabs* infection. Indeed, infection of classical immunocompetent mouse models leads to transient colonization, thus impeding their use as a valuable animal models to study chronic disease and the *in vivo* therapeutic efficacy of drugs (Bernut et al., 2014b). However, in the past few years the development of multiple cellular, non-mammalian and mammalian models have helped to study the chronology and the pathology of *Mabs* infection (Byrd and Lyons, 1999; Howard et al., 2006; Ordway et al., 2008; Oh et al., 2013; Bernut et al., 2014a; Bakala N'Goma et al., 2015; Roux et al., 2016). Among these new model systems, a few have been validated for their suitability for *in vivo* drug efficacy studies against *Mabs* (Ordway et al., 2008; Lerat et al., 2014; Oh et al., 2014; Bernut et al., 2014b). These different infection models and their applications are discussed below in more details.

### Cellular models to understand early infection with *M. abscessus*

**Figure 1:**
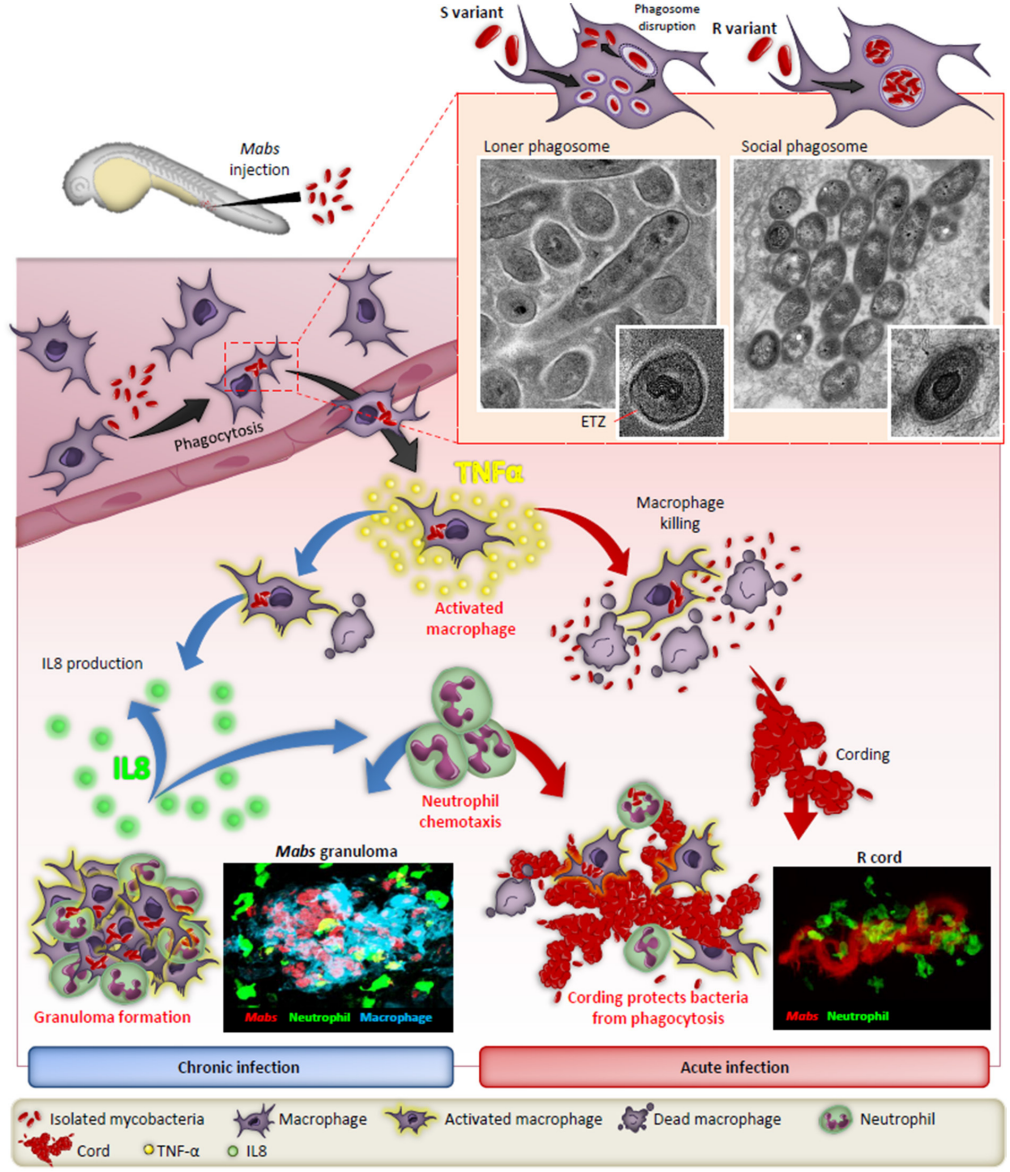
**The distinct fates of the smooth (S) and rough (R) variants of ***M. abscessus*** in macrophages and zebrafish**. After injection in the blood flow, *Mabs* are rapidly phagocytozed by macrophages, either individually (S variant) in loner phagosomes or as bacterial clusters (R variant) in social phagosomes. The presence of GPL production in the S strain leads to a typical electron translucent zone (ETZ) that fills the entire space between the phagosome membrane and the mycobacterial cell wall. Loss of GPL in the R variant results in disappearance of the ETZ. In some instances, disruption of the phagosome membrane releases the S variant in the cytoplasm. Infected macrophages migrate from the vasculature the nervous tissues and become heavily infected which leads to apoptosis with the release of the S variant that are phagocytosed by newly recruited macrophages which, together with neutrophils, form protective granulomas, resulting in chronic infection. In contrast, the release of *Mabs* R is correlated with the emergence of extracellular serpentine cords, preventing phagocytosis of the bacilli by macrophages and neutrophils, leading to abscess formation with tissue destruction and acute infection. TNF-α plays a central role in the immunity to *Mabs* by activating the macrophage bactericidal response and, through IL-8 production, in neutrophil chemotaxis to the site of infection or to form protective granulomas. Adapted from Bernut et al. (2016).

Studies using murine, human primary macrophages or cell lines allowed for the description of early invasion of *Mabs* S and R phenotypes inside phagocytic cells. Initially, the S phenotype establishes a more silent phase while the R phenotype forms a more aggressive infiltration in cells (Byrd and Lyons, 1999). A major hallmark of *Mabs* R is its high propensity to aggregate, leading to the formation of phagocytic cups and the presence of social phagosomes containing usually more than one bacillus. These phagocytic cups and bacterial laden phagosomes are associated with increased cell mortality, evoking a typical trait of RGM (Brambilla et al., 2016; Roux et al., 2016). In contrast, loner phagosomes containing one bacilli are usually found within macrophages infected with the S form (Figure 1). These studies also reported the sustained intramacrophage survival of the S variant over the R variant and the increased resistance of the S variant toward cellular bactericidal mechanisms, such as phago-lysosomal fusion block and resistance to apoptosis and autophagy. The S variant-containing phagosome shows signs of membrane alteration, such as breaks or partial degradation at a very early stage of the infection, further emphasizing the ability of *Mabs* S to initiate phagosome-cytosol communications (Roux et al., 2016). In contrast to *M. tuberculosis*, this phagosomal escape mechanism is ESX-1-independent as *Mabs* does not possess an ESX-1 homolog (Ripoll et al., 2009). Overall, these phenotypic features resemble more to those characterizing pathogenic slow-growing mycobacteria than RGM. The blocking of autophagic clearance by azithromycin, a drug administered to CF patients as an anti-inflammatory compound, leads to the intracellular survival of *Mabs* (Renna et al., 2011).

Foamy macrophages have been proposed as a reservoir used by *M. tuberculosis* for long-term persistence within its human host (Peyron et al., 2008). They represent a granuloma-specific cell population characterized by their high lipid content. An experimental model of foamy macrophages was recently designed to investigate acquisition of host lipids by pathogenic mycobacteria contained within phagosomes (Caire-Brändli et al., 2014; Santucci et al., 2016). This cellular model, derived from murine bone-marrow macrophages, has recently been exploited to investigate the formation of intracellular lipid inclusion (ILI) in *Mabs* and to demonstrate that, like *M. tuberculosis, Mabs* can accumulate triglycerides under the form of large ILI inside foamy macrophages (Viljoen et al., 2016). These cells allowed to demonstrate the ability of *Mabs* to assimilate host lipids and the crucial role of the diacylglycerol acyltransferases Dgat1 in ILI formation (Viljoen et al., 2016), which may represent an important source for long-term storage and energy supply enabling persistence.

CF lungs are characterized by an important neutrophilic inflammation in response to pathogens, such as *Staphylococcus aureus* or *Pseudomonas aeruginosa*. However, internalization of *Mabs* by neutrophils appears less efficient as compared to *S. aureus*, explained by the fact that *Mabs* limits neutrophil activation which promotes pathogen survival, highlighting the abilities of *Mabs* to adapt to harsh immune environments (Malcolm et al., 2013). Like most mycobacteria, *Mabs* exploits its surface-exposed lipids to interact directly with the macrophage pattern recognition receptors. Among these receptors, Toll Like Receptor (TLR-2) and Dectin-1 have been shown to participate in phagocytosis of *Mabs* (Shin et al., 2008; Rhoades et al., 2009; Roux et al., 2011). Interestingly, the transition from an S to an R phenotype is associated with robust inflammation, resulting from the loss of GPL which is unmasking bacterial surface exposed TLR-2 agonists, such as lipoproteins (Roux et al., 2011). In addition, Jönsson et al. (2013) studied the response of human peripheral blood mononuclear cells (PBMC) to *Mabs* R infection identifying a fibrous meshwork comprised of DNA and histones surrounding the *Mabs* cords. This suggests that the chromatin meshwork may represent a defense mechanism against *Mabs* invaders. Conversely, *Mabs* S, which are unable to form cords, result in rapid phagocytosis and failed to induce similar fibrous structures in PBMC.

### Exploiting amoebae to identify new virulence determinants of *M. abscessus*

Exploring the interaction between *Mabs* and eukaryotic cells has significantly contributed to delineate the basis of the early interaction and survival strategies between the pathogen in its human host. Although *Mabs* is regarded as an emerging human pathogen, *Mabs* is primarily an environmental opportunistic microorganism. It has been proposed that free-living amoebae, which provide an intracellular niche similar to phagocytes, are functioning as a training ground for most environmental NTM (Drancourt, 2014). This has recently stimulated studies aimed at describing interactions between *Mabs* and *Ancathamoeba castellanii*, an amoeba used as a model species to study the interaction with many different microorganisms (Guimaraes et al., 2016). Co-cultures have shown the induced expression of several *Mabs* virulence determinants, such as the phospholipase C or MgtC when *Mabs* is present inside the amoeba (Bakala N'Goma et al., 2015; Le Moigne et al., 2016). Additionally, a *Mabs* R mutant defective in cording showed severely impaired replication in both macrophages and amoebae, presumably due to limited inhibition of the phagolysosomal fusion. These studies emphasize the usefulness of amoebae as a host model to identify and study determinants important in sustaining *Mabs* virulence (Halloum et al., 2016). Despite the failure of isolating *Mabs* from environmental sources, studies with *Acanthamoeba* suggest that *Mabs* has evolved in close contact with environmental protozoa and support the view that amoebae contribute in shaping *Mabs* virulence (Bakala N'Goma et al., 2015; Le Moigne et al., 2016).

### *M. abscessus* infection in the zebrafish model

Zebrafish (*Danio rerio*) have been widely exploited during the last two decades to decipher the intricate interactions between pathogens and the host immune system (Torraca et al., 2014). The increasing success of this vertebrate model relies on unique advantages that motivated its use to increase our knowledge of many viral and bacterial infections (Davis et al., 2002; Prajsnar et al., 2008; Phennicie et al., 2010; Alibaud et al., 2011; Mostowy et al., 2013; Palha et al., 2013). Zebrafish embryos have also recently been exploited to visualize, by non-invasive imaging, the progression and fate of *Mabs* infection, allowing to observe host-pathogen interactions in a live animal at a high resolution level (Bernut et al., 2015; Figure 1). Methods were specifically adapted for assessing *Mabs* virulence by measuring distinct parameters, such as embryo survival and bacterial burden and by monitoring the chronology of the infection using video microscopy. These studies culminated with the description and visualization of extracellular cording characterizing the R variant as a new mechanism of immune subversion by preventing the bacilli from phagocytosis by macrophages and neutrophils, thus promoting infection and rapid larval death (Bernut et al., 2014a). Moreover, cords were also found to initiate the formation of abscesses, mainly disseminated within the central nervous system (brain and spinal cord). These initial observations have stimulated further work to shed new light on the role of cording in pathogenesis. This has led to the recent identification of the *MAB_4780* gene encoding a dehydratase. Disruption of this gene in *Mabs* R was associated with the lack of granuloma formation in embryos and a highly attenuated phenotype in wild-type and in embryos lacking either macrophages or neutrophils (Halloum et al., 2016). Overall, work using the zebrafish model confirmed the crucial role of MAB_4780 for *Mabs* cording to successfully establish acute and lethal infections. In addition, in studies evaluating a loss-of-function coupled with fluorescent reporter zebrafish lines and high resolution imaging, the contribution of TNF-mediated signaling to protective immunity against *Mabs* has been confirmed (Bernut et al., 2016). Furthermore, identifying the crucial role of TNF in activating macrophage bactericidal activity leading to restriction of intracellular *Mabs* growth and IL8-mediated neutrophil recruitment for development and maintenance of protective granulomas was demonstrated (Figure 1). However, despite their unique features, major disadvantages of zebrafish embryos over mammalian models, resides in their anatomical differences, such as gills instead of lungs. In addition, the lack of adaptive immunity early in the development may also impact the outcome of the infection. In general, embryos appear more suited to study acute infection rather than the chronic stages of the disease, which are better modeled in mice.

### The mouse model of *M. abscessus* lung infection

Early studies confirmed that most immunocompetent mouse strains result in clearance of *Mabs* in the first weeks after infection with *Mabs* isolates (Ordway et al., 2008; Bernut et al., 2014b; Obregón-Henao et al., 2015), making model development and selection challenging. In these earlier studies, C57BL/6 and leptin-deficient (Ob/Ob) mice infected with *Mabs* with a low-dose aerosol inoculum (LDA, ~100 bacilli per mouse) did not develop a sustained progressive infection. Conversely, when infected with a high-dose aerosol inoculum (HDA, ~1,000 bacilli per mouse), C57BL/6 and Ob/Ob mice established an infection resulting in an early influx of IFN-γ^+^ CD4^+^ T cells in the lungs. This primary immune response preempted the clearance of *Mabs* in both C57BL/6 and Ob/Ob mice. On the other hand, IFN-γ knockout (GKO) mice infected with a LDA or HDA of *Mabs* resulted in a low amount of persistent *Mabs* lung infection inducing influx of T cells, macrophages and dendritic cells, which contributed to granuloma formation. Interestingly, a HDA *Mabs* infection of the GKO mice provoked CD4^+^ and CD8^+^ T cells capable of producing IL-4 and IL-10 in the pulmonary cavity.

Despite the aforementioned challenges associated with establishing a high level of *Mabs* infection, further research has led to establishment of mouse models with deficits in innate or acquired immunity, resulting in a high level of *Mabs* disease. Initial studies established that immunocompromised mice with defects in innate or acquired immunity infected intravenously with 1 × 10^6^ *Mabs* controlled the infection (Obregón-Henao et al., 2015). Mouse strains able to clear *Mabs* include Beige (dominant T_H_2 immunity), iNOS^−/−^, Cybb^−/−^ (devoid of super-oxide generating enzyme), TNFαR^−/−^, C3HeB/FeJ, GKO, and MyD88^−/−^ mice. Through a 40 days chronic infection, *Mabs* was still present at low levels in the lungs of the C3HeB/FeJ, GKO and MyD88^−/−^ mice. Furthermore, the GKO and MyD88^−/−^ mice sustained diminished amounts of *Mabs* in the spleen and liver after 40 days, whereas SCID, nude and GM-CSF^−/−^ mice infected intravenously with *Mabs* showed progressive *Mabs* burden (Rottman et al., 2007; Obregón-Henao et al., 2015), which revealed the requirement of functional T and B cell immunity and GM-CSF reliant cell phenotypes for establishment of protective immunity against *Mabs*. It is clear that deleting single genes for NOS, ROS, TNF, IFNγ, and MyD88 alone results in other immune mechanisms compensating for their loss, resulting in low levels of *Mabs* persistence or complete removal of *Mabs*. However, multiple deficits in innate and acquired immunity result in a high level *Mabs* progressive infection. A major advantage of using severely immunocompromised mice (SCID, nude, and GM-CSF^−/−^) for modeling *Mabs* infection was the presence of foamy cells and necrotizing and non-necrotizing granulomas in the lungs after 40 days of infection, commonly observed in the histopathologic sections of human NTM lung disease. The challenge remains to overcome RGM avirulence and immune clearance in immunocompetent models for future research advances. Multiple CF models have also been developed for other human CF pathogens. Current studies are focused on developing these CF models for *Mabs* infection advancing into the development of pulmonary infection rather than using the intravenous route.

### Preclinical models to assess drug efficacy against *M. abscessus*

**Figure 2:**
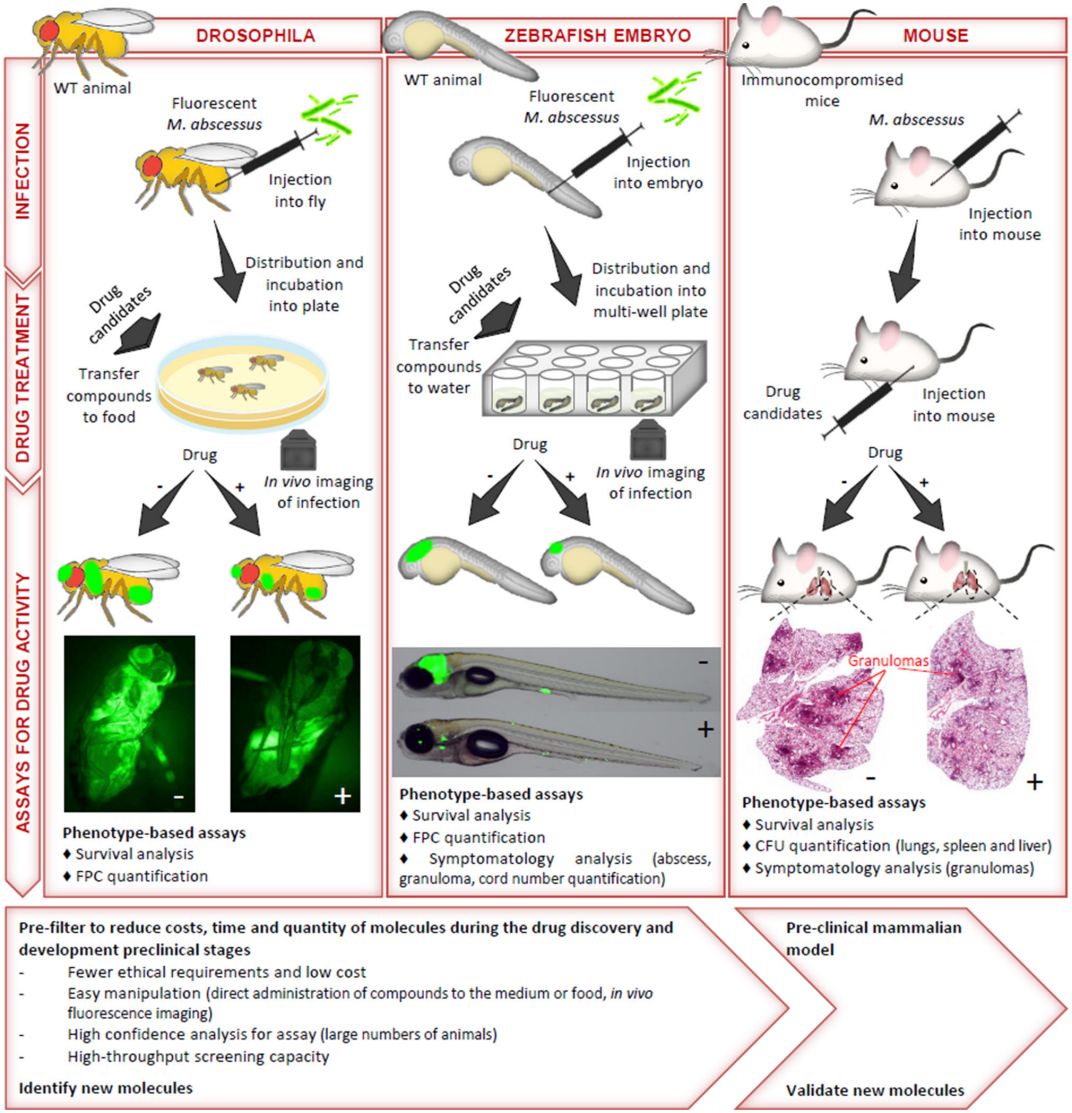
**The most commonly used preclinical models to study the ***in vivo*** activity of compounds against ***M. abscessus*****. The methods of infection, the drug treatment conditions and the assays used to monitor drug activity of anti-*Mabs* molecules in drosophila, zebrafish embryos and immunocompromised mice are illustrated. The advantages and limitations of each model, from the early stages of drug screening to assessments of the preclinical efficacy of the more advanced compounds are also indicated. CFU, colony-forming unit; FPC, fluorescent pixel count.

Due to the intrinsic and acquired resistance mechanisms of *Mabs* to most commonly used antimicrobials, the discovery of new and active compounds is urgently needed. A major step in drug discovery relies on *in vivo* evaluations of the identified hits, requiring studies in adequate animal models. As already mentioned, immunocompetent mice are characterized by a transient infection with a rapid clearance of *Mabs* (Bernut et al., 2014b), thus impeding their use as valuable animal models for drug susceptibility testing. Alternative immunocompromised models have, therefore, been developed (Figure 2). GM-CSF knock-out mice have been shown to recapitulate chronic pulmonary *Mabs* infection and to be particularly suited for preclinical drug efficacy testing (De Groote et al., 2014). In particular, exposure of *Mabs*-infected GM-CSF KO mice to azithromycin resulted in reduced bacterial loads in the lungs and spleen and weight gain with significant improvement in lung pathology. Nude mice have also been proposed as appropriate for *in vivo* drug efficacy assessments (Lerat et al., 2014). The anti-*Mabs* activity of multiple drugs have been tested for efficacy in both the GKO and SCID mice model (Obregón-Henao et al., 2015). Drug efficacy in ascending order were clarithromycin, clofazamine, amikacin, bedaquiline, and clofazamine-bedaquiline. The treatment of *Mabs* infected SCID mice with a combination of clofazamine-bedaquiline resulted in increased efficacy against *Mabs* compared to the other drug regimens.

However, despite the fact that immunocompromised mice present a significant advance in comparison to wild-type mice in preclinical assessments, they remain costly, time-consuming and may not reflect the predictive value required for compound testing. Zebrafish were also successfully developed to test the suitability and sensitivity of clarithromycin and imipenem, two clinically relevant drugs in *Mabs*-infected embryos (Bernut et al., 2014b). One major advantage of this model is that it allows to visualize in a dose- and time-dependent manner the resorption/disappearance of cords and abscesses in the presence of an active molecule (Figure 2). The demonstration of the efficacy of a combination consisting of a β-lactam (amoxicillin or imipenem) and a *Mabs* β-lactamase specific inhibitor (avibactam) further validated the zebrafish as a potent preclinical model (Dubée et al., 2015; Lefebvre et al., 2017). Recently, the *in vivo* activity of a new piperidinol-based compound, PIPD1, inhibiting mycolic acid transport in *Mabs* was evaluated in zebrafish (Dupont et al., 2016). Exposure to PIPD1 increased embryo survival and reduced the bacterial burden and the number of abscesses. Moreover, because zebrafish allows to mimic a CF micro-environment by silencing *cftr* expression (Phennicie et al., 2010), this biological system would be suited at comparing the therapeutic efficacy of drugs in a *cftr*-deficient environment as it is currently not known whether a CFTR defect affects susceptibility to antibiotics. Of note, embryos are particularly conducive to high throughput screening, as shown for *Mycobacterium marinum* (Carvalho et al., 2011; Takaki et al., 2012), which may speed up the process of identifying promising drug candidates. Another advantage of this model is the ease and rapidity of experimentation within a restricted time scale and low cost. In this context, *Drosophila melanogaster* has also been reported for the rapid evaluation of potential drug candidates against *Mabs* (Oh et al., 2014), offering the advantages of speed, cost, technical convenience and ethical acceptability (Figure 2).

## Conclusion and perspectives

Herein, we have summarized compelling models developed to better understand the interplay between *Mabs*, its environmental and human hosts. These biological systems have undoubtedly allowed to better elucidate the pathogenesis of *Mabs* disease and to highlight the distinctive intra- and extracellular traits characterizing the lifestyle of the R and S forms. However, despite considerable progress, our knowledge of the immunopathology of *Mabs* infection remains largely incomplete. As discussed here, these recently developed models represent innovative tools for better manipulating both the pathogen and the host immune response and should lead to an in-depth comprehension of the intricate interactions between the pathogen and its hosts, which may also unveil novel strategies to combat the infection and disease progression. Although several challenges still need to be overcome, these models hold also great promises for future development of novel and improved drug combinations to control one of the most difficult-to-treat mycobacterial species.

## Author contributions

All authors contributed to writing the manuscript. AB designed the figures.

### Conflict of interest statement

The authors declare that the research was conducted in the absence of any commercial or financial relationships that could be construed as a potential conflict of interest.
